# Alterations in dopamine system and in its connectivity with serotonin in a rat model of Alzheimer’s disease

**DOI:** 10.1093/braincomms/fcab029

**Published:** 2021-03-10

**Authors:** Kelly Ceyzériat, Yesica Gloria, Stergios Tsartsalis, Christine Fossey, Thomas Cailly, Frédéric Fabis, Philippe Millet, Benjamin B Tournier

**Affiliations:** 1 Division of Adult Psychiatry, Department of Psychiatry, University Hospitals of Geneva, 1206 Geneva, Switzerland; 2 Division of Nuclear medicine, Diagnostic Department, University Hospitals and Geneva University of Geneva, 1206 Geneva, Switzerland; 3 Division of Radiation Oncology, Department of Oncology, University Hospitals of Geneva, 1206 Geneva, Switzerland; 4 Normandie University, UNICAEN, Centre d’Etudes et de Recherche sur le Médicament de Normandie (CERMN), 14000 Caen, France; 5 Department of Nuclear Medicine, CHU Cote de Nacre, 14000 Caen, France; 6 Normandie University, UNICAEN, IMOGERE, 14000 Caen, France; 7 Department of Psychiatry, University of Geneva, Geneva, Switzerland

**Keywords:** Alzheimer’s disease, D_2_ receptor, 5HT_2A_ receptor, amyloid, TSPO

## Abstract

Dopamine pathways alterations are reported in Alzheimer’s disease. However, it is difficult in humans to establish when these deficits appear and their impact in the course of Alzheimer’s disease. In the TgF344-Alzheimer’s disease rat model at the age of 6 months, we showed a reduction in *in vivo* release of striatal dopamine due to serotonin 5HT_2A_-receptor blockade, in the absence of alterations in 5HT_2A_-receptor binding, suggesting a reduction in 5HT_2A_-receptor-dopamine system connectivity. In addition, a functional hypersensitivity of postsynaptic dopamine D_2_-receptors and D_2_-autoreceptors was also reported without any change in D_2_-receptor density and in the absence of amyloid plaques or overexpression of the 18 kDa translocator protein (an inflammatory marker) in areas of the dopamine system. Citalopram, a selective serotonin reuptake inhibitor, induced functional 5HT_2A_-receptor−D_2_-receptor connectivity changes but had no effect on D_2_-autoreceptor hypersensitivity. In older rats, dopamine cell bodies overexpressed translocator protein and dopamine projection sites accumulated amyloid. Interestingly, the 5HT_2A_-receptor density is decreased in the accumbens subdivisions and the substantia nigra pars compacta. This reduction in the striatum is related to the astrocytic expression of 5HT_2A_-receptor. Our results indicate that both serotonin/dopamine connectivity and dopamine signalling pathways are dysregulated and potentially represent novel early diagnostic and therapeutic avenues.

## Teaser

Ceyzériat et al. demonstrate dysfunction in serotonin/dopamine connectivity and functional hypersensitivity of dopamine receptors in the TgF344-AD rat model of Alzheimer’s disease aged 6 months. Neurochemical alterations in serotonin receptor were also reported at an advanced age. Consequently, these results suggest dopamine and serotonin systems as new early-diagnostic and therapeutic avenues.

## Graphical Abstract

**Figure:**
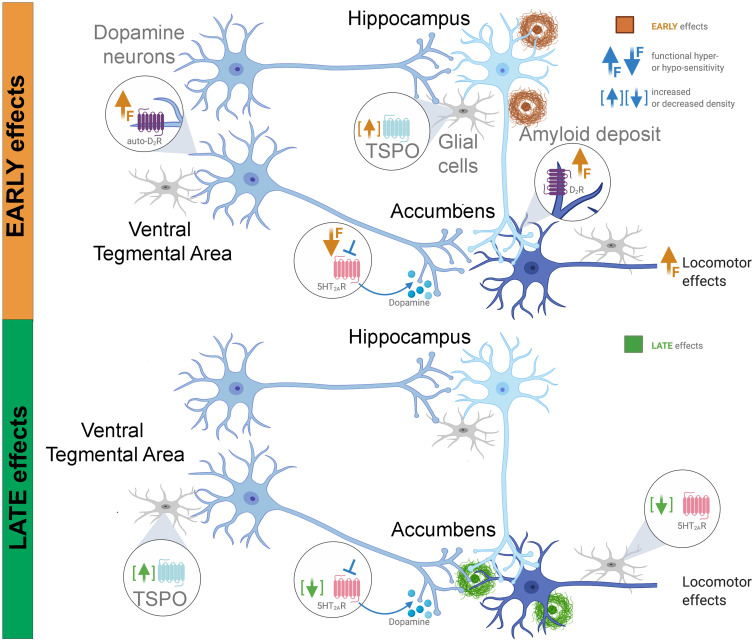

## Introduction

In addition to the major pathological hallmarks of Alzheimer’s disease, including the presence of Aβ deposits, neurofibrillary tangles, neuroinflammation and brain atrophy, some reports seem to indicate that earlier events could to some extent be indicators of the progression towards the development of pathology. Among them have been described the presence of neuropsychiatric symptoms which may appear before clinical manifestations of dementia and are linked to the loss of capacity of patients to perform instrumental activities of daily living.^1^^,^^2^ The possibility of dopaminergic dysfunction has been suggested as an actor in these behavioural disorders.^1^ In this sense, post-mortem studies have shown a reduction in dopamine (DA) synthesis,^3^ an alteration of the neurons producing DA and DA release in the striatum and hippocampus,^4^ but the impact on DA D_2_-receptor (D_2_R) density is not clear.^5–7^ More than half of the patients develop behavioural disorders related to the DA system.^8^ It is even possible that these alterations play a role in the onset of memory disorders, due to the involvement of DA in cognitive processes.^9^^,^^10^

In addition, other monoaminergic systems are deregulated in the disease. This is particularly the case with the serotonin (5HT) system, with for example a decrease in 5HT 2A-receptor (5HT_2A_R) density.^11^ Interestingly, there is a significant cross-talk between the activity of the DA system and that of the 5HT system. Indeed, for example, the 5HT_2A_R is expressed by DA neurons,^12^^,^^13^ DA release is influenced by the 5HT_2A_R activity^14–16^ and the control of locomotor activity is at least partially dependent on the D_2_R and 5HT_2A_R activity.^17–19^ Thus, the presence of a functional impairment of 5HT or DA could also result in functional alteration of the other neuroregulatory system.

However, in humans it is difficult to determine the sequence of events. The direct link between DA/5HT alterations and the neuropsychiatric disorders observed in Alzheimer’s disease patients is not clearly demonstrated. One study described early deficits in serotoninergic transmission in MCI patients which correlate with depression and anxiety symptoms.^20^ However, more studies are necessary to better understand the role of DA/5HT alterations in Alzheimer’s disease and to determine if these alterations are early events compared to the molecular hallmarks of Alzheimer’s disease and consequently appear before the expansion of amyloid plaques, neurofibrillary tangles and inflammation, or conversely if they are a consequence. It is also likely that an alteration of one of these two systems induces an impairment of the function of the other. Indeed, DA signalling pathway could be functionally altered by a change in 5HT_2A_R/DA connectivity.^18^ The highlighting of functional and neurochemical alterations is important in order to better characterize the pathology with the aim of carrying out early diagnoses and detecting new opportunities for therapeutic pathways. Indeed, while clinical trials targeting amyloid plaques, Tau pathology or inflammation have been disappointing,^21^ it seems fundamental to better understand the evolution of the pathology from the early stages and find ways to characterize initial deficits.

In this study, we characterized the dopaminergic function linked to the activity of D_2_-autoreceptors and postsynaptic D_2_R and deficits in the connectivity between the 5HT_2A_R function and the DA system. To do this, we used the TgF344-AD rat model of Alzheimer’s disease. In this model, the age of 6 months can be considered cognitively pre-symptomatic since the rats do not show cognitive impairment (with the exception of one study that did report a cognitive impairment at this age) while they present some amyloid pathology, low levels of soluble Tau and an intraneuronal Tau expression restricted to the locus coeruleus.^22–26^ At this age, we used functional *in vivo* SPECT imaging to measure both D_2_R availability and DA release. As the ventral striatum is mainly involved in the mesolimbic pathway for motivation control and the dorsal striatum in the nigrostriatal pathway for movement control, these two striatal subregions were analysed separately. Then, given that at the age of 6 months, TgF344-AD rats do not present any cognitive deficit, we have used well described tests of dopaminergic reactivity in order to characterize any functional deficits of the DA system. Notably, we used tests of locomotion as marker of this activity. Thus, we characterized the locomotor responses to the stimulation of presynaptic or postsynaptic D_2_R alone or in presence of 5HT_2A_R inhibition. In addition, we tested the effect of chronic citalopram treatment, a selective serotonin reuptake inhibitor (SSRI), on D_2_R and 5HT_2A_R function. Finally, we examined these results with regard to neurochemical alterations in 18 kDa translocator protein (TSPO), amyloid deposits, D_2_R and 5HT_2A_R densities observed at a pre-symptomatic stage and at a more advanced stage, using the well described [^125^I]CLINDE,^27^^,^^28^ [^125^I]DRM106,^29–31^ [^125^I]Epidepride^32^^,^^33^ and [^125^I]R91150^34^^,^^35^ radioligands, respectively.

## Materials and methods 

### Animals

Male TgF344-AD rats harbouring APP_SWE_ and PS1_ΔE9_ transgenes (TgAD in figures) and their WT littermates were used at 6 and 18 months of age. Animals were kept on 12‐h light/dark cycle and had *ad libitum* access to water and food. All experimental procedures were approved by the Ethics Committee for Animal Experimentation of the Canton of Geneva, Switzerland.

### Citalopram treatment

Six-month-old TgF344-AD rats were exposed to the SSRI citalopram (Sigma, Switzerland) in the drinking water (0.3 mg/ml) during 10 consecutive weeks (*n* = 7). Control animals (WT and TgF344-AD rats) were given only water (*n* = 7/group). Behavioural tests (locomotion and elevated plus maze) were performed on the last week of the citalopram/water treatment. Considering a mean water consumption of 8–11 ml/100 g of body weight,^36^ the estimation of citalopram consumption is around 30 mg/kg/day. In rats, this dose delivered by drinking water has been shown to induce comparable plasma levels to the human therapeutic range and normalize behavioural and neurochemical effects induced in a chronic stress model.^37^^,^^38^

### *In vivo* SPECT imaging

The *in vivo* release of DA in the striatum in response to an injection of a 5HT_2A_R antagonist was previously demonstrated using the measurement of D_2/3_R radioligand binding inhibition.^16^ Herein, we used a similar approach with the 5HT_2A_R antagonist MDL100.907 and the D_2/3_R radioligand [^125^I]IBZM. Under isoflurane anaesthesia, animals (*n* = 6/group) received intravenous injections of [^125^I]IBZM at *t*_0_ (WT: 30.6 ± 3.5 MBq; TgAD: 30.9 ± 3.6 MBq) and *t*_115_ min (WT: 31.0 ± 3.0 MBq; TgAD: 30.7 ± 3.6 MBq) and MDL100.097 (0.1 mg/kg) at *t*_110_ min. Two 30-min scans were acquired using U-SPECT-II (MILabs, Utrecht, The Netherlands), 80 min after the first and the second [^125^I]IBZM injection, which correspond to *t*_80 − 110_ and *t*_195 − 225 _min. Images were reconstructed using a POSEM (0.4 mm voxels, four iterations, six subsets) approach, with radioactive decay correction applied. Image processing was done using PMOD software (PMOD Technologies Ltd, Zurich, Switzerland). Following a manual co-registration to the rat MRI implemented in the software, a volume-of-interest template was used to extract the radioactivity measurements. A specific binding ratio (SBR) was obtained by normalizing the activity images in ventral and dorsal striatum to the activity in the cerebellum minus one. DA release was estimated as follows: Δ[^125^I]IBZM = (SBR second injection × 100/SBR first injection) − 100.

### Locomotor activity test

An open-field was used to measure locomotor activity in response to saline, presynaptic (0.05 mg/kg) and postsynaptic (0.5 mg/kg) doses of the D_2_-preferring agonist quinpirole^39^ and to test the impact of a pre-treatment with the 5HT_2A_R antagonist MDL100.907. The apparatus consists of 4 square boxes of 45×45×40 cm overlooked by a digital camera. The distance travelled was automatically analysed by the Noldus software (EthoVision, Noldus).

Three different cohorts performed a 3-days test, with two injections per day. These three groups were used to test the impact of the presynaptic dose of quinpirole, the impact of the postsynaptic dose of quinpirole and the effect of the SSRI treatment on the locomotor response to the presynaptic dose of quinpirole. Thus, the first cohort (WT and TgF344-AD rats, *n* = 8/group) received saline-saline on Day 1, saline-quinpirole (0.05 mg/kg) on Day 2 or 3 and MDL100.907 (0.1 mg/kg)-quinpirole on Day 3 or 2 (according to a Latin square design), 40 and 10 min before a 30-min recording, respectively. The second cohort (WT and TgF344-AD rats, *n* = 8/group) received saline-saline on Day 1, saline-quinpirole (0.5 mg/kg) on Day 2 or 3 and MDL100.907 (0.1 mg/kg)-quinpirole on Day 3 or 2 (according to a Latin square design), 40 and 30 min before a 60-min recording, respectively. The last cohort (WT, TgF344-AD rats and SSRI-treated TgF344-AD rats, *n* = 8/group) performed the same locomotor recordings as the first cohort (i.e. saline-saline on Day 1, saline and 0.05 mg/kg quinpirole on Day 2 or 3 and 0.1 mg/kg MDL100.907 and quinpirole on Day 3 or 2, according to a Latin square design, 40 and 10 min before a 30-min recording, respectively).

### Elevated plus-maze test

Animals were placed in the centre of the apparatus (4 arms of 50×10 cm, two of them with 50 cm walls, connected by a central square of 10×10 cm) and recorded for 5 min (*n* = 7/group). Noldus software was used to calculate the number of head-dipping (the animal tilts its head over the arm to observe the ground) and the total duration of head-dipping behaviour as an anxiety index.

### *In situ* autoradiography

To estimate the densities of TSPO, D_2/3_R, 5HT_2A_R and amyloid deposits, *in situ* autoradiography was performed as previously described^31^ using [^125^I]CLINDE, [^125^I]Epidepride, [^125^I]R91150 and [^125^I]DRM106, respectively (*n* = 8–9/group). The synthesis of the CLINDE stannylated precursor (2‐{6‐Chloro‐2‐[4‐(tributylstannyl)phenyl]imidazo[1,2‐*a*]pyridin‐3‐yl}‐*N*,*N*‐diethylacetamide) was performed in 4 steps from 4‐(4‐bromophenyl)‐4‐oxobutanoic acid (see Supplementary material for details). Iododestannylation performed with I_2_ on the CLINDE stannylated precursor allowed the formation of the cold reference for the CLINDE radiosynthesis.

The radiosynthesis of [^125^I]CLINDE, [^125^I]R91150 and [^125^I]DRM106 has been described in detail in previous papers.^31,40–43^ The general procedure is identical to the one used herein for the synthesis of [^125^I]Epidepride, as follows. The epidepride precursor was incubated for 30 min with Na^125^I (185–370 MBq, PerkinElmer), 1 μl of 30% H_2_O_2_ and 1 μl acetic acid. Diluted reaction with 50% acetonitrile (ACN) was then purified using a linear gradient HPLC run (5−95% ACN in 7 mM H3PO4, 10 min) with a reversed-phase column (Bondclone C18). Brain samples were immerged with 0.11 MBq/ml of radiotracer with specific activity was greater than 650 GBq/μmol for all of the radiotracers, based on the limit of detection of the ultraviolet absorbance and on the calibration curves established with cold reference compounds.

The tissue preparation was performed as previously reported.^31^ Briefly, animals were transcardially perfused with 0.9% saline and their brain quickly removed, frozen at −30°C in pre-cooled isopentane and stored at −80°C until use. Serial coronal brain sections (20 µm) were cut on a cryostat and slices were immersed in a Tris−MgCl_2_ buffer (50 mM Tris−HCl, 50 mM MgCl_2_, pH 7.4) alone (20 min), then in the same buffer containing either [^125^I]CLINDE, [^125^I]Epidepride, [^125^I]R91150 or [^125^I]DRM106 (0.11 MBq/ml, 90 min) then rinsed twice in 4°C buffer (3 min) and briefly washed in cold water. For [^125^I]DRM106, buffers also contained 20% EtOH. Non-specific binding was estimated in the presence of 10 µM of unlabelled ligands on adjacent sections. Air-dried slides were then exposed to gamma-sensitive phosphor imaging plates (Fuji BAS-IP MS2325) and resulting autoradiograms were analysed with Aida Software V4.06 (Raytest Isotopenmessgerate GmbH) together with homemade ^125^I calibration curves. To determine regions-of-interest (ROI), brain sections were stained for acetylcholinesterase and used as histological reference. Nineteen ROI were selected (see details in figures). The results corresponding to the ROIs of the entire accumbens (core and shell subregions) are not shown, as specific ROI for accumbens core and ROI for accumbens shell displayed identical data. The SBR was calculated as follows: (ROI/ROI with 10 μM of unlabelled radiotracer) – 1. For each animal and targets, four to eight sections were analysed and values in bilateral ROI were averaged.

### Fluorescence-activated cell sorting to radioligand-treated tissues

To quantify the [^125^I]R91150 binding on striatal astrocytes and microglia, the fluorescence-activated cell sorting to radioligand-treated tissues (FACS-RTT) approach was used.^44^ Animals were euthanized 60 min after an intravenous [^125^I]R91150 injection (44.05 ± 1.4 MBq) (*n* = 7–11/genotype). Different brain regions were dissected (frontal cortex, striatum, hippocampus and the cerebellum) on ice, weighted and measured on a γ-counter. Striatal cells were dissociated by mechanical and enzymatic actions as described in detail previously.^44^ Dissociated cells were sorted by FACS (Beckman Coulter MoFlo Astrios) according to a positive and negative selection protocol. Negative selection excluded autofluorescent cells and positive cells for the FITC anti-rat CD90 (neural marker, 1/250; Biolegend) and PE-Cy7 anti-rat CD31 (endothelial marker, 1/70; Invitrogen) antibodies. Positive selection allowed positive cells to be retained separately for the antibodies Cy3 anti-GFAP (astrocytic marker, 1/12; Sigma) and APC anti-rat CD11b (microglial marker, 1/800; Biolegend). The number of cells was measured during cell sorting. Radioactivity in astrocytic and microglial cell populations was then measured using a γ-counter and data were expressed as % of the injected dose.

### Statistical analysis

A power size analysis (Douglas Altman’s nomogram) was performed prior to randomly assign rats in their treatment group. One-way repeated measures ANOVA with brain region as within-subject factor and genotype as between-subject factor was used to analyse SPECT [^125^I]IBZM binding, fold change in [^125^I]IBZM binding, *in situ* autoradiography*, ex vivo* [^125^I]R91150 binding. One-way repeated measures ANOVA with pre/post treatment period as within-subject factor and brain area as between-subject factor was used to analyse MDL100.907-induced changes in [^125^I]IBZM binding. The locomotor behaviour was analysed using a three-way ANOVA with time, genotype and treatment as between-subject factors. When considering the total recording period, a two-way ANOVA was used (genotype and treatment). Two-way ANOVA with arm and group as factors was used to analyse localization of rats in the EPM. One-way ANOVA (group effect) was used to analyse the head-dipping behaviour in the EPM. When ANOVA analyses revealed significant main effects or interaction effects a planned comparison including only the difference between groups/treatment and not the entire contrasts were used to assess *post hoc* comparisons using LSD *post hoc* test. When only two groups are compared (number of astrocytes/microglia, [^125^I]R91150 binding in astrocytes/microglia) a two-tailed Student’s *t*- tests was used. Data are indicated as mean ± SEM.

### Data availability

Raw data can be accessed at https://doi.org/10.26037/yareta:Zxsf4cx4yvgtxc2kn2jefm3xde

## Results

### Functional deficiencies in the 5HT_2A_R-dopamine/D_2_R connectivity at the age of 6 months

**Figure 1:**
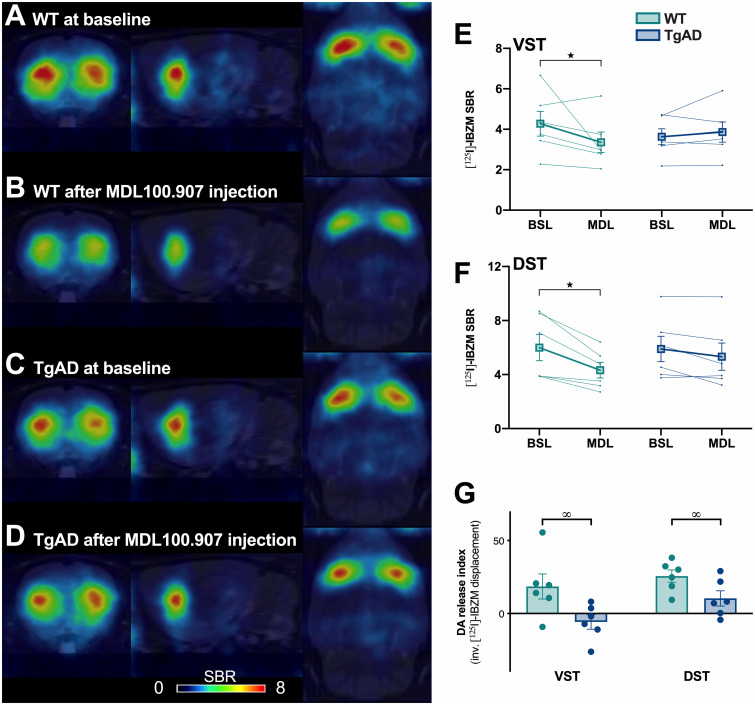
**MDL100.907-induced dopamine release in the striatum is decreased in 6-month-old TgF344-AD rats.** D_2/3_R density at baseline and dopamine release in response to the i.v. injection of MDL100.907 was tested in 6-month-old rats by in vivo SPECT imaging using [^125^I]IBZM (*n* = 6/genotype). **(A−D)** Representative images of the SPECT [^125^I]IBZM signal at the level of the striatum. Images were coregistered to the MRI atlas in the coronal (left), sagittal (centre), and horizontal (right) planes. **(E** and **F)** [^125^I]IBZM binding at baseline (BSL) and following the injection of MDL100.907 (MDL) in ventral (VST) and the dorsal (DST) striatum. **(G)** DA release index (inverse of the displacement of the [^125^I]-IBZM binding between the two SPECT acquisitions, after and before MDL100.907 injection) in ventral (VST) and the dorsal (DST) striatum. Significant main effects of sub-regions (#), MDL100.907 injection (★) and genotype (∞) are indicated as *P < 0.05.

To measure if the 5HT_2A_R control of dopaminergic activity is altered in TgF344-AD rats, a translational *in vivo* imaging test to measure D_2/3_R density and DA release was carried out by SPECT using [^125^I]IBZM. Mean parametric images of WT and TgF344-AD rats at baseline and following the i.v. injection of the 5HT_2A_R antagonist MDL100.907 are presented in Fig. 1A−D. At baseline, no difference between genotypes on the [^125^I]IBZM binding was observed, but a sub-region effect was pointed out (Fig. 1E and F). Indeed, the dorsal striatum showed a significant higher D_2/3_R density than the ventral striatum, in accordance with literature [two-way ANOVA, genotype: *F*_(1,5)_ = 0.55, *P* > 0.05; striatal subregion: *F*_(1,5)_ = 8.71, *P* < 0.05; genotype × striatal subregion interaction: *F*_(1,5)_ = 2.09, *P* > 0.05; LSD *post hoc* test for striatal subregion: *P* < 0.05].

The decrease in [^125^I]IBZM binding between the second and the first scan acquisition, indicating an increase in DA release, was measured in dorsal and ventral striatum (Fig. 1E−G). In WT, the MDL100.907 injection induced a reduction in the [^125^I]IBZM binding in dorsal (−25.6 ± 4.23%) and ventral striatum (−18.5 ± 8.61%), demonstrating a significant increase in DA release independently of the striatal subregion [one-way repeated ANOVA, MDL treatment: *F*_(1,5)_ = 7.79, *P* < 0.05; striatal subregion: *F*_(1,5)_ = 6.43, *P* = 0.052; MDL treatment × striatal subregion interaction: *F*_(1,5)_ = 2.99, *P* > 0.05]. In contrast, the effect of MDL100.907 treatment seems to impact differentially the ventral (+5.8 ± 4.97%) and the dorsal (−10.3 ± 5.2%) striatum in TgF344-AD rats, that may indicate a preferential alteration in the mesolimbic than the nigrostriatal pathway [one-way repeated ANOVA, MDL treatment: *F*_(1,5)_ = 0.6, *P* > 0.05; striatal subregion: *F*_(1,5)_ = 6.58, *P* = 0.0503; MDL treatment × striatal subregion interaction: *F*_(1,5)_ = 12.07, *P* < 0.05; LSD *post hoc* test for the interaction: *P* > 0.05]. The direct comparison of the fold change in [^125^I]IBZM binding due to the MDL100.907 injection between WT and TgF344-AD rats indicated that DA release was significantly lower in ventral and dorsal striatum of TgF344-AD compared to WT rats (Fig. 1G). However, no difference between striatal subregions is observed [two-way ANOVA, genotype: *F*_(1,5)_ = 8.59, *P* < 0.05; striatal subregion: *F*_(1,5)_ = 3.47, *P* > 0.05; genotype × striatal subregion interaction: *F*_(1,5)_ = 2.63, *P* > 0.05; LSD *post hoc* test for genotype: *P* < 0.05].

### Functional hyper-sensitivity to presynaptic D_2_R stimulation at the age of 6 months

To evaluate a change in the reactivity of the DA system in 6-month-old TgF344-AD rats, we used locomotor tests in response to the stimulation of auto-D_2_R or post-synaptic D_2_R.

**Figure 2:**
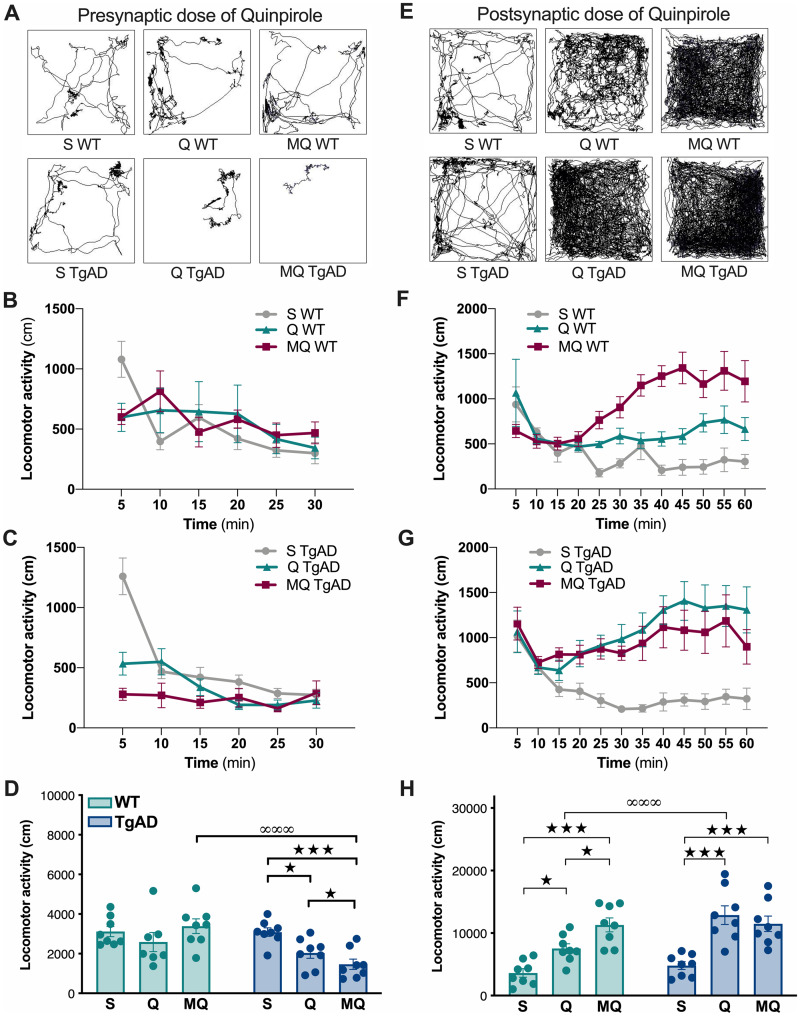
**Quinpirole-induced locomotor inhibition and activation is increased in 6-month-old TgF344-AD rats.** The locomotor response to a presynaptic (0.05 mg/kg, **A−D**) and a postsynaptic (0.5 mg/kg, **E−H**) dose of quinpirole alone or in association with a MDL100.907 pretreatment is presented in WT and TgF344-AD rats at 6 months of age (*n* = 8/group). (**A**, **E**) Representative example of behavioural pattern in response to saline and pre- or post-synaptic doses of quinpirole in WT and TgF344-AD rats. (**B, C)** Locomotor activity (in 5 min blocks) in response to saline (S), presynaptic dose of quinpirole (Q) or MDL100.907/quinpirole (MQ) in WT (**B**) and TgF344-AD (TgAD) rats (**C**). (**D**) Total distance travelled, in response to saline (S), quinpirole (Q) or MDL100.907/quinpirole (MQ) in WT and TgF344-AD (TgAD) rats using pre-synaptic doses of quinpirole. (**F**, **G**) Locomotor activity (in 5 min blocks) in response to saline (S), postsynaptic dose of quinpirole (Q) or MDL100.907/quinpirole (MQ) in WT (**F**) and TgF344-AD (TgAD) rats (**G**). (**H)** Total distance travelled, in response to saline (S), quinpirole (Q) or MDL100.907/quinpirole (MQ) in WT and TgF344-AD (TgAD) rats using postsynaptic doses of quinpirole. Significant main effects of treatments (★) and genotype (∞) are indicated as *P < 0.05; ***P < 0.001.

The locomotion inhibitory effect induced by a low dose of quinpirole (0.05 mg/kg) has been associated with presynaptic D_2_R stimulation.^39^ Thus, WT and TgF344-AD rats received either saline or a low (0.05 mg/kg) dose of quinpirole to measure the functional sensitivity of presynaptic D_2_R. Representative examples of locomotor patterns are given in Fig. 2A.

The locomotor response to the low dose of quinpirole alone or in association with MDL100.907 pretreatment is presented in as 5-min interval locomotion recording in WT (Fig. 2B) and TgF344-AD rats (Fig. 2C). We observed a different effect of quinpirole on WT and TgF344-AD rat behaviour, and this independently of the time interval observed [three-way ANOVA, genotype × quinpirole interaction: *F*_(1,168)_ = 4.67, *P* < 0.05, genotype × quinpirole × time interaction: *F*_(5,168)_ = 0.21, *P* > 0.05]. Analysis of the locomotor response to MDL100.907 at 5-min interval locomotion did not reveal interaction with the time interval [three-way ANOVA, genotype: *F*_(1,168)_ = 30.9, *P* < 0.001; genotype × treatment interaction: *F*_(1,168)_ = 1.38, *P* > 0.05, genotype × treatment × time interaction: *F*_(5,168)_ = 0.75, *P* > 0.05]. Thus, we considered the total recording period to compare the effect of treatment in each group and between groups (Fig. 2D). Quinpirole alone or in association with the MDL100.907 pretreatment, effectively impacted differently WT and TgF344-AD rat locomotion [two-way ANOVA, treatment main effect: *F*_(2,41)_ = 3.77, *P* < 0.05, genotype: *F*_(1,41)_ = 10.7, *P* < 0.01; treatment × genotype interaction: *F*_(2,41)_ = 5.02, *P* < 0.05]. Quinpirole alone or Quinpirole + MDL did not alter locomotion in WT rats [LSD *post hoc* test: *P* > 0.05], but reduced it significantly in TgF344-AD rats (*P* < 0.05), suggesting a presynaptic hyper-sensitivity to quinpirole as compared to WT. In addition, the MDL100.907 pretreatment enhanced the inhibitory effect of quinpirole in TgF344-AD rats (*P* < 0.05). This results in lower locomotor activity in TgF344-AD rats than in controls in response to the co-injection of MDL100.907/quinpirole (*P* < 0.001).

The analysis of the first 10 min of the locomotor recordings conducted to the same conclusions (data not shown).

### Functional hyper-sensitivity to postsynaptic D_2_R stimulation at the age of 6 months

The locomotion activating effect induced by a higher dose of quinpirole (0.5 mg/kg) has been associated with postsynaptic D_2_R activation.^39^ Thus, WT and TgF344-AD rats received either saline or a high (0.5 mg/kg) dose of quinpirole to measure the functional sensitivity of postsynaptic D_2_R. Representative examples of locomotor patterns are given in Fig. 2E.

The locomotor response to the high dose of quinpirole alone or in combination with MDL100.907 is presented in as 5-min interval locomotion recording in WT (Fig. 2F) and TgF344-AD rats (Fig. 2G). We observed a different effect of quinpirole depending on the genotype but not on the time interval studied [three-way ANOVA: genotype × quinpirole interaction: *F*_(1,305)_ = 26.6, *P* < 0.001; genotype × quinpirole × time interaction: *F*_(11,305)_ = 0.98, *P* > 0.05]. In contrast, the locomotor response to the addition of MDL100.907 to postsynaptic dose of quinpirole is dependent of the time interval [genotype × treatment interaction: *F*_(1,336)_ = 21.38, *P* < 0.001, genotype × treatment × time interaction: *F*_(11,336)_ = 2.45, *P* < 0.01]. Considering the total recording period (Fig. 2H), we can notice a distinct locomotor response of WT and TgF344-AD animals to drug injections [two-way ANOVA, treatment main effect (saline, quinpirole, MDL100.907 + quinpirole): *F*_(2,42)_ = 27.4, *P* < 0.001; genotype: *F*_(1,42)_ = 9.95, *P* < 0.05; drug × genotype interaction: *F*_(2,42)_ = 3.48, *P* < 0.05]. The postsynaptic dose of quinpirole significantly stimulated locomotion in both WT [LSD *post hoc* test: *P* < 0.05] and TgF344-AD rats (*P* < 0.001). However, the stimulated locomotor activity was significantly greater in TgF344-AD rats than in controls (*P* < 0.001), indicating a hyper-sensitivity to postsynaptic D_2_R stimulation. In addition, the MDL100.907 pretreatment enhanced the stimulatory effect of quinpirole in WT rats (*P* < 0.05) but was ineffective in TgF344-AD rats.

### Chronic SSRI-mediated 5HT pathway stimulations improve behaviours

**Figure 3:**
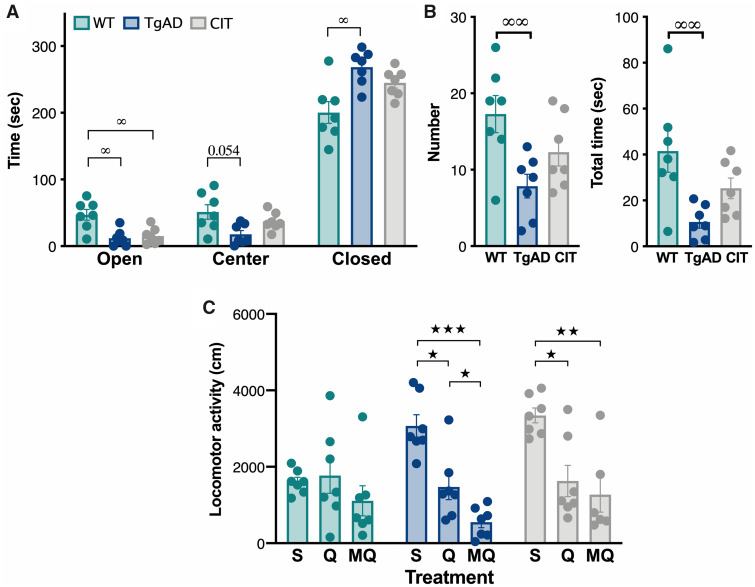
**Chronic SSRI treatment decreases anxiety levels and reduces locomotor inhibition in 6-month-old TgF344-AD rats.** Untreated WT and TgF344-AD (TgAD) rats were compared to chronically citalopram treated TgAD (CIT) in terms of anxiety in the elevated plus maze (**A**, **B**) and quinpirole/MDL100.907-induced locomotor inhibition (**C**) (*n* = 7/group). (**A**) Time spent in the centre and open and closed arms of the elevated plus maze. (**B)** Number and total duration (s) of head-dipping (the animal tilts its head over the arm to observe the ground) in the elevated plus maze. (**C**) Locomotor response to saline (S), pre-synaptic quinpirole (Q) or MDL100.907/pre-synaptic quinpirole (MQ). Significant effects of treatments (★) and genotype (∞) are indicated as *P < 0.05; **P < 0.01; ***P < 0.001.

To determine the effectiveness of chronic 5HT stimulation in modifying locomotor response to dopamine and dopamine/serotonin stimulation, animals received a 10-week citalopram treatment in drinking water. In order to validate the efficacy of the treatment in modifying serotonin-related behaviours, anxiety levels were measured in the EPM test (Fig. 3A and B). A tendency to a reduction of the time spent in the centre (*P* = 0.054), an increase in the time spent in closed arms (*P* < 0.05) and a decrease in the time spent in open arms (*P* < 0.05) was reported in TgF344-AD vs WT rats, reflecting an increased anxiety in untreated AD rats [two-way ANOVA, genotype × area interaction: *F*_(4,36)_ = 9.47, *P* < 0.001; LSD *post hoc* test results are indicated in brackets]. No difference was observed between citalopram-treated TgF344-AD and WT rats in the time spent in the centre and closed arms of the EPM, but the time spent in the open arms is still higher for WT than citalopram-treated TgF344-AD rats (*P* < 0.05, Fig. 3A). A decrease of head-dipping behaviour, a complementary index to evaluate anxiety like-behaviour,^45^^,^^46^ was also observed in TgF344-AD rats compared to controls, confirming the higher anxiety in AD rats (Fig. 3B) [one-way ANOVA, group effect for number of head-dipping (*F*_(2,18)_ = 5.77, *P* = 0.0116), LSD *post hoc* test: *P* < 0.01; group effect for total head dipping duration (*F*_(2,18)_ = 6.37, *P* < 0.01), LSD *post hoc* test: P  < 0.01). Importantly, the head dipping number performed by TgF344-AD rats is no longer significantly different from WT after the citalopram treatment, suggesting that citalopram reduced anxiety-like behaviours. However, as values of citalopram-treated rats appeared at intermediary levels with no difference between either WT or TgF344-AD, it can be suggested that there is an ameliorative effect of the treatment but not a full restoration of anxiety levels.

The locomotor activity in response to drugs injection was different between WT, TgF344-AD and citalopram-treated rats (Fig. 3C) [two-way ANOVA, treatment × group interaction: *F*_(4,53)_ = 3.55, *P* = 0.0122; treatment: *F*_(2,53)_ = 19.84, *P* < 0.001]. While WT rats were not sensitive to drugs [LSD *post hoc* test: *P* > 0.05], control TgF344-AD rats showed a reduced locomotor activity in response to presynaptic quinpirole (*P* < 0.05) that is enhanced by MDL100.907 injection (*P* < 0.05), confirming the previous experiment. The SSRI treatment in TgF344-AD rats did not suppress quinpirole-induced locomotor decrease (*P* < 0.05), but it inhibited the potentiating effect of MDL100.907 injection. The comparison of locomotor activity in TgF344-AD with and without SSRI treatment did not show any difference (*P* > 0.05).

### Absence of alterations in D_2/3_R and 5HT_2A_R density at the age of 6 months

To better understand the neurochemical basis of functional D_2/3_R and 5HT_2A_R alterations in TgF344-AD rats, a quantification of their densities in the brain was performed. This analysis was extended to two Alzheimer’s disease’s markers, amyloid and inflammation, by quantifying amyloid plaques and TSPO.

**Figure 4:**
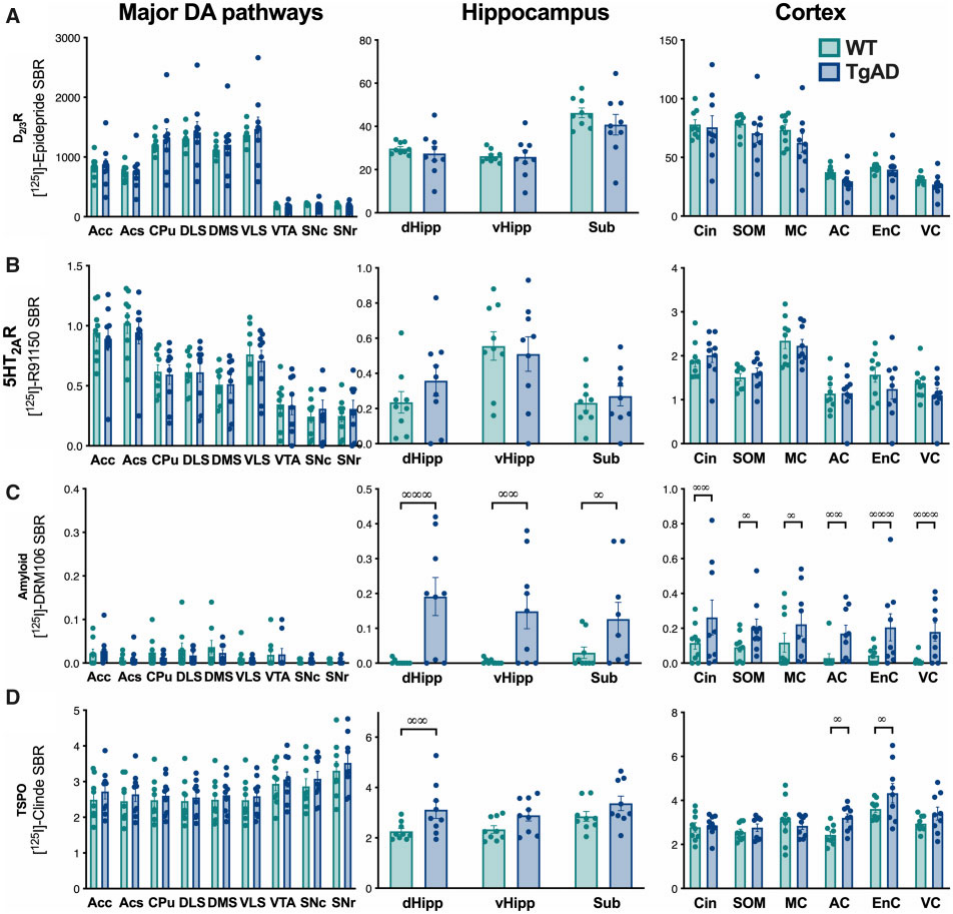
**Amyloid and TSPO are observed in hippocampus and cortex areas in 6-month-old TgF344-AD rats.** The evaluation of the density in D_2_R (**A**) and 5HT_2A_R (**B**), amyloid deposits (**C**) and TSPO (**D**) was carried out by *in situ* autoradiography with [^125^I]Epidepride and [^125^I]R91150, [^125^I]DRM106 and [^125^I]CLINDE, respectively (*n* = 9/group). Measurement were performed in the major DA pathways (*left column*), hippocampus areas (*centre*) and in cortex (*right column*). Genotype effects (*post hoc* test) are indicated as ∞: *P* < 0.05; ∞∞: *P* < 0.01 and ∞∞∞: *P* < 0.01. AC, auditory cortex; Acc, accumbens core; Acs, accumbens shell; Cin, cingulate cortex; CPU, caudate/putamen; dHipp, dorsal hippocampus; DLS, dorsolateral striatum; DMS, dorsomedial striatum; EnC, enthorinal cortex; MC, motor cortex; SNc, substantia nigra pars compacta; SNr, substantia nigra pars reticulata; SOM, somatosensorial cortex; Sub, subiculum; VC, visual cortex; vHipp, ventral hippocampus; VLS, ventrolateral striatum; VTA, ventral tegmental area.

At 6 months old, TgF344-AD rats showed the same densities as the controls in terms of D_2/3_R as of 5HT_2A_R (Fig. 4A and B) [two-way ANOVA, D_2/3_R: genotype: *F*_(1,16)_ = 0.068, *P* > 0.05; region: *F*_(18,288)_ = 187, *P* < 0.001; genotype × region interaction: *F*_(18,288)_ = 0.3, *P* > 0.05; 5HT_2A_R: genotype: *F*_(1,16)_ = 0.068, *P* > 0.05, region: *F*_(18,288)_ = 108, *P* < 0.001, genotype × region interaction: *F*_(18,288)_ = 1.07, *P* > 0.05].

Amyloid deposits are absent in the dopaminergic system regions, but are already detectable in the hippocampus (subiculum and both dorsal and ventral hippocampi) and in all the cortices analysed (Fig. 4C) [two-way ANOVA, genotype: *F*_(1,16)_ = 7.29, *P* < 0.05; region of the brain: *F*_(18,288)_ = 8, *P* < 0.00; genotype × region interaction: *F*_(18,288)_ = 3.18, *P* < 0.001].

Interestingly, TSPO accumulation differ between WT and TgF344-AD depending on the region studied (Fig. 4D) [two-way ANOVA, genotype effect: *F*_(1,16)_ = 1.56, *P* > 0.05; brain region: *F*_(18,288)_ = 14.5, *P* < 0.001; genotype × region interaction: *F*_(18,288)_ = 1.96, *P* < 0.05]. A higher presence of TSPO in TgF344-AD vs WT rats was detected in the dorsal hippocampus [LSD *post hoc* test: *P* < 0.01], the entorhinal cortex (*P* < 0.05) and the auditory cortex (*P* < 0.05).

### 5HT_2A_R density are reduced in some dopaminergic and cortical areas at the age of 18 months

To determine if at a more advanced pathological stage (i.e. represented by an older age) an alteration of the densities in D_2/3_R or 5HT_2A_R appears, a quantification by *in situ* autoradiography in the brain of 18-month old rats was performed.

**Figure 5:**
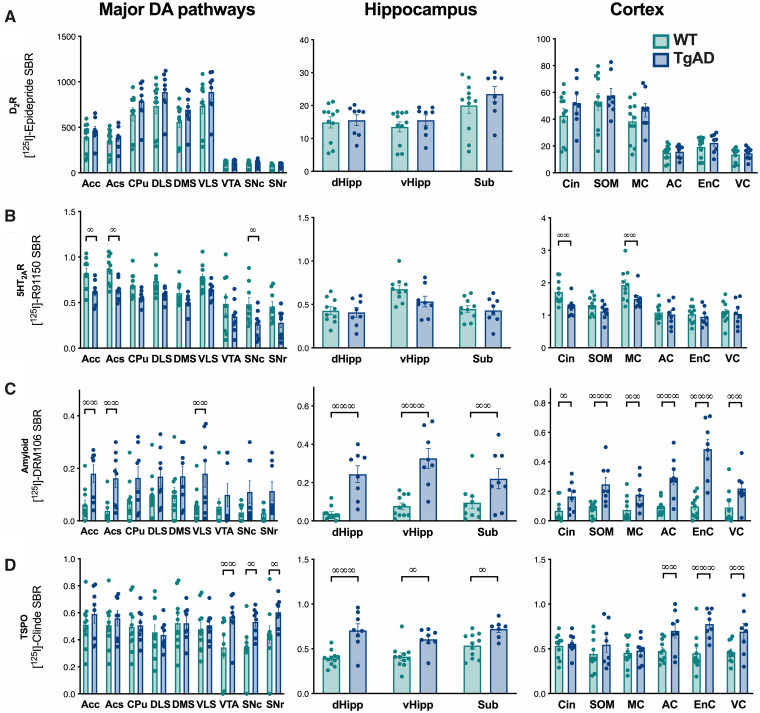
**Increases in amyloid and TSPO and a decrease in 5HT_2A_R density are observed in 18-month-old TgF344-AD rats.** The evaluation of the density in D_2_R (**A**) and 5HT_2A_R (**B**), amyloid deposits (**C**) and TSPO (**D**) was carried out by *in situ* autoradiography with [^125^I]Epidepride and [^125^I]R91150, [^125^I]DRM106 and [^125^I]CLINDE, respectively (*n* = 8/group). Measurement were performed in the major DA pathways (*left column*), hippocampus areas (*centre*) and in cortex (*right column*). Genotype effects (*post hoc* test) are indicated as ∞: *P* < 0.05; ∞∞: *P* < 0.01 and ∞∞∞: *P* < 0.01. AC, auditory cortex; Acc, accumbens core; Acs, accumbens shell; Cin, cingulate cortex; CPU, caudate/putamen; dHipp, dorsal hippocampus; DLS, dorsolateral striatum; DMS, dorsomedial striatum; EnC, enthorinal cortex; MC, motor cortex; SNc, substantia nigra pars compacta; SNr, substantia nigra pars reticulata; SOM, somatosensorial cortex; Sub, subiculum; VC, visual cortex; vHipp, ventral hippocampus; VLS, ventrolateral striatum; VTA, ventral tegmental area.

D_2/3_R density was not different between 18-month-old control and TgF344-AD rats (Fig. 5A) [two-way ANOVA, genotype: *F*_(1,16)_ = 0.699, *P* > 0.05; brain region: *F*_(18,288)_ = 196, *P* < 0.001; genotype × brain region interaction: *F*_(18,288)_ = 0.98, *P* > 0.05].

In contrast, a significant region-dependent decrease in 5HT_2A_R density is measured in TgF344-AD rats (Fig. 5B) [two-way ANOVA, genotype: *F*_(1,16)_ = 6.09, *P* < 0.05; brain region: *F*_(18,288)_ = 77.6, *P* < 0.001; genotype × brain region interaction: *F*_(18,288)_ = 1.96, *P* < 0.05]. This reduction in 5HT_2A_R density concerns one DA neurons cell bodies’ region (substantia nigra pars compacta, LSD *post hoc* test: *P* < 0.05), DA neurons projections’ regions (accumbens core and shell, *P* < 0.05), the cingulate and motor cortex (*P* < 0.01).

At 18-month-old, a significant accumulation of amyloid deposits in the DA neurons projection regions (accumbens core and shell, ventrolateral striatum) and, as at 6-month-old, in the hippocampus and cortical areas was observed (Fig. 5C) [two-way ANOVA, genotype: *F*_(1,16)_ = 18.3, *P* < 0.001; brain region: *F*_(18,288_) = 9.15, *P* < 0.001; genotype × brain region interaction: *F*_(18,288)_ = 5.15, *P* < 0.001].

TSPO over-expression was also extended compared to the age of 6 months and concerned dopaminergic system regions [substantia nigra pars compacta and reticulata, ventral tegmental area (VTA)], the hippocampus (subiculum, dorsal and ventral hippocampus) and some cortices (auditory, entorhinal and visual; Fig. 5D) [two-way ANOVA, genotype: *F*_(1,16)_ = 6.46, *P* < 0.05; region: *F*_(18,288)_ = 2.92, *P* < 0.001; genotype × region interaction: *F*_(18,288)_ = 3.08, *P* < 0.001].

### 5HT_2A_R reduction in striatal astrocytes of 18-month-old TgF344 AD rats

**Figure 6:**
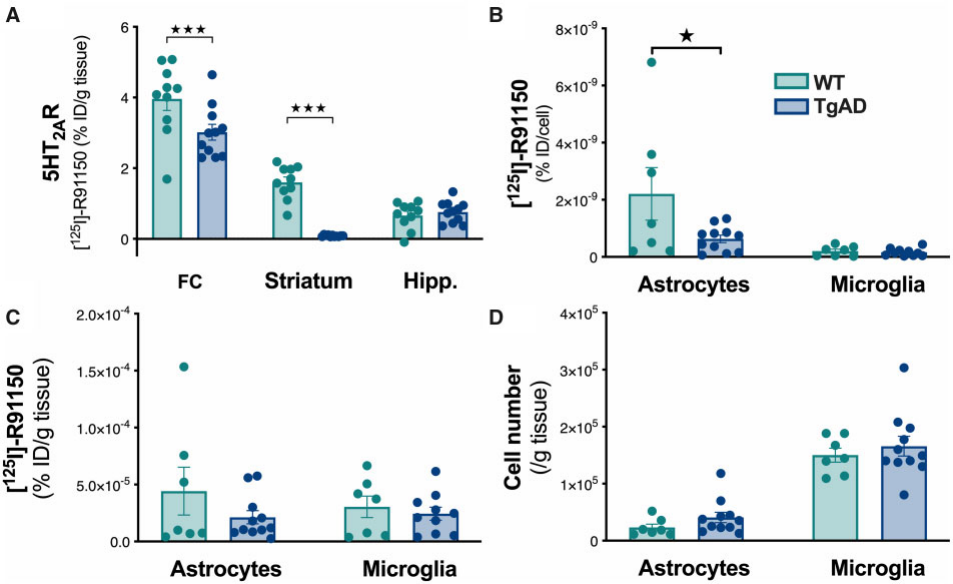
**5HT_2A_R is decreased in astrocytes of the striatum in 18-month-old TgF344-AD rats.** The [^125^I]R91150 concentration was determined in brain areas (%injected dose/g of tissue, **A**), in astrocytes and microglia at cell (%injected dose/cell, **B**) and at cell population level (%injected dose/g of tissue, **C**) levels. The number of cells sorted was quantified (**D**); *n* = 7–11/genotype, ^★^*P* < 0.05; ^★★★^*P* < 0.001. FC, frontal cortex; Hipp., hippocampus.

To further characterize the reduction in 5HT_2A_R density, a quantification in glial cells was performed in the total isolated striatum. The *ex vivo* binding measured over the entire parenchyma showed a decrease in 5HT_2A_R density depending on the region (Fig. 6A) [two-way ANOVA, genotype: *F*_(1,19)_ = 15.38, *P* < 0.001; region: *F*_(2,38)_ = 277, *P* < 0.001; genotype × region interaction: *F*_(2,38)_ = 18.8, *P* < 0.001]. Indeed, we observed reduced levels in the frontal cortex (main tissue for the 5HT_2A_R expression) and the striatum but not in the hippocampus, confirming the *in situ* autoradiography study.

To selectively isolate microglial cells and astrocytes from the striatum, FACS technic was used. CD90+ cells containing the neuronal cell bodies were excluded during the cell isolation protocol, dendrites like axons are partly cut off which would have affected the precise determination of 5HT_2A_R density. The number of 5HT_2A_R per astrocyte is significantly decreased (Fig. 6B) [*t*(16) = 2.12, *P* < 0.05]. This effect is neither associated with any change at the cell population level [*t*(16) = 1.27, *P* > 0.05] nor with alterations in the number of astrocytes [*t*(16) = 1.5, *P* > 0.05] (Fig. 6C and D).

However, no difference was observed between TgF344-AD and WT rats for microglial cells [radioactivity at cell population level: *T*(16) = 0.61, *P* > 0.05; at cell level: *T*(16) = 0.61, *P* > 0.05; number of microglial cells: *T*(16) = 0.45, *P* > 0.05].

## Discussion

Clinical trials targeting Aβ and phosphorylated-Tau in Alzheimer’s disease have had discouraging results.^21^ It thus seems important to seek new therapeutic routes and biomarkers for earlier diagnosis. In this idea, it is important to note that approximately one patient out of two presents alterations of the dopaminergic system activity which could take part in the clinical decline.^1^^,^^8^^,^^47^ In our study with the TgF344-AD rat, a model of transgene-induced amyloid accumulation and endogenous Tau pathology,^22^ we show that functional dysfunction in DA pathways appear early and that 5HT_2A_R-mediated control of DA/D_2_R function is also altered. Our study, using the translational SPECT imaging approach shows that the functional 5HT/DA connectivity is altered in rats at an age before the onset of cognitive symptoms.^24^^,^^48–50^ At an advanced stage of the pathology, we also show 5HT_2A_R density alterations in dopamine areas and cortical regions. Thus, data, combined with the evidence of DA^10^^,^^47^^,^^51^ and 5HT^52^^-^^54^ neurotransmitter system involvement in cognitive impairment suggests that dysfunctions of these monoaminergic pathways could participate in the pathophysiology of memory impairment in Alzheimer’s disease.

Dopaminergic system dysfunction has been reported in Alzheimer's disease patients.^8^ Neuropsychiatric symptoms appear early in some patients and could represent a diagnostic marker of the pathology.^1^^,^^47^ At the cellular level, the presence of D_2/3_R density alterations has not been reproduced across the various studies.^4–6^^,^^55–59^ However, the use of antagonist ligands for measuring D_2/3_R density does not allow to distinguish the active from the inactive forms of these receptors.^60^ In the case of D_2_R in particular, the presence of an active D2^high^ (‘high-affinity’), and an inactive, D2_low_ (‘low-affinity’), form has been shown.^61^ It is thus possible that in the absence of a difference in total D_2_R, the distribution between the active D2^high^ form and the inactive D2_low_ form is altered in TgF344-AD rats. This hypothesis could explain the hypersensitivity of D_2_R, and future studies will have to answer this question. Moreover, the activity of DA pathways is under the control of various systems including the 5HT system. Among its receptors, 5HT_2A_R has been shown to be expressed by cortical neurons projecting into the striatum, at the level of SN/VTA, and also by DA neurons.^12^^,^^13^^,^^62^^,^^63^ The blocking of its intrinsic activity by antagonists, such as MDL100.907, induces DA release including in the striatum.^14–16^ Our data in WT animals agree with this observation. Interestingly, we showed that at the age of 6 months DA release is significantly reduced in TgF344-AD rats in response to 5HT_2A_R blockage. This suggests an alteration of 5HT_2A_R−DA connectivity.

In order to better characterize the 5HT_2A_R−D_2_R connectivity dysfunction, MDL100,907 was co-administered with either a presynaptic or a postsynaptic dose of quinpirole. The combined treatment with MDL100,907 and a presynaptic quinpirole dose led to a reduced locomotor activity in the TgF344-AD rats, compared to the quinpirole treatment alone. This observation suggests an amplification of the effect of D_2_-autoreceptor stimulation in the presence of a 5HT_2A_R blockade. The absence of a synergistic effect between the injection of MDL100.907 and the stimulating postsynaptic dose of quinpirole in TgF344-AD supports a decrease in 5HT_2A_R functioning, considering the MDL100.907-induced striatal DA release effect. In contrast, in WT rats, the MDL100.907 injection acts synergistically with the postsynaptic dose of quinpirole which leads to an increase in locomotion vs. quinpirole alone. Thus, this decrease in 5HT_2A_R−DA system connectivity could also reflect a more generally decrease in 5HT_2A_R functioning. In addition, the two doses of agonist used assume that this hyper-reaction concerns both D_2_-autoreceptors and postsynaptic D_2_R.

Interestingly, the functional hypersensitivity of D_2_R and the dysfunction in 5HT_2A_R−D_2_R connectivity are detected without changes in DA and 5HT_2A_ receptor density at 6-month-old rats. At this age, we already observed the presence of Aβ deposits in the hippocampus and in the studied cortical areas. It has been shown that 6-month-old rats did not show clear cognitive impairments yet.^22^ This finding underlines the interest of studying possible dopaminergic/serotoninergic functional alterations appear early in human MCI and Alzheimer’s disease. Moreover, at this age, TSPO inflammation is still restricted to the dorsal hippocampus and few areas of the cortex. Neuroanatomical links connect the VTA to the hippocampus and the hippocampus to the accumbens, which implies a strong interconnectivity between the hippocampus and the DA system. For example, hippocampal activity has been shown to influence DA release in the striatum^64^ and addiction-related behaviours.^65^ It is interesting to note that the functional DA changes observed here are present at an age when the hippocampus showed TSPO increases and amyloid accumulation. Inflammation^66^ as well as amyloid^67^^,^^68^ has been shown to induce alterations in neuronal conductivity. It therefore appears possible that early hippocampal dysfunction contributes to the appearance of functional DA disorders by an alteration of hippocampus−DA relationships. The recent report showing a reduction in the participation of TgF344-AD rats in behavioural tests also supports the presence of a deficit in the dopaminergic system.^50^ As it was previously reported that SSRI treatment can alter the activity of DA neurons,^69^ we tested the impact of chronic exposure to citalopram on the response to quinpirole alone or in combination with MDLl00.907. The SSRI treatment appeared to be effective on reducing anxiety levels, and on the control of 5HT_2A_R on the locomotor response to presynaptic quinpirole, but does not allow a normalization of the hyper-sensitivity of D_2_-autoreceptors. Taken together, our data support the hypothesis that functional 5HT disorders predate anatomical observations, which implies that the cellular environment or neural networks are modified by the pathology and could ultimately lead to cell death and reduction in the number of 5HT_2A_R.

To validate this hypothesis, we also analysed D_2/3_R and 5HT_2A_R levels at an age when the pathology is more advanced. At the age of 18 months TgF344-AD rats present behavioural disorders, reduced cognitive capacities and endogenous tauopathy.^22^^,^^49^ We effectively observed a significant decrease in 5HT_2A_R density in some cortical and DA system areas. In patients, a decrease in 5HT_2A_R has also been reported and may be associated with the severity of the cognitive deficit.^52^^,^^70^^,^^71^ Even if the complete mechanism of this finding is not understood, it could be associated with the presence of soluble Αβ.^52^ At the pre-symptomatic stage, this 5HT_2A_R loss was not observed even if amyloid plaques were already present. Thus, it is possible that the increase in the most toxic forms of the amyloid with age in TgF344-AD rats^22^ induces this alteration. In the mesolimbic pathway, we observed a decrease in 5HT_2A_R in the accumbens but not in the cell DA soma in VTA. In addition, in the nigrostriatal pathway, SNc showed a decreased level in 5HT_2A_R but not in the subregions of the dorsal striatum. Thus, both nigrostriatal and mesolimbic pathways may be affected by a change in the 5HT_2A_R−DA connectivity. This hypothesis agrees with the reduction in DA release in both ventral and dorsal striatum in response to 5HT_2A_R blockade. However, 5HT_2A_R reduction in SNc did not appear to be a consequence of local Aβ accumulation as SNc did not showed increases in amyloid. In contrast, the accumbens showed both amyloid accumulation and 5HT_2A_R reduction that may, at least in part, explain why the alteration in DA release in accumbens tends to be stronger than in the dorsal striatum. Importantly, as astrocytes and microglia also express 5HT_2A_R^52^^,^^72^^,^^73^, we explored and identify a reduction in striatal 5HT_2A_R in astrocytes where 5HT_2A_R play a role in the regulation of the activity of astrocytes.^74^^,^^75^ This reduction could therefore assume a reduced effect of the control by 5HT of the activity of astrocytes, including in the control of the astrocytic regulation of dopaminergic function.^76^ It is also possible that such an effect was present in the others brain regions affected by the 5HT_2A_R density reduction. As striatal astrocyte’s activity contributes to the proper functioning of the DA system,^76–79^ and considering the activator role of 5HT_2A_R in Ca^2+^ signalling in astrocytes,^74^ it is possible that a decrease in astrocytic 5HT_2A_R alters their functions. In line with this idea, the astrocytes of the accumbens respond to DA and participate in the control of locomotion.^76^ Thus, the decrease in 5HT_2A_R-mediated function in young animals could be amplified by 5HT_2A_R loss in old animals. This disappearance of 5HT_2A_R density could lead to impaired functions linked to 5HT_2A_R activity including its role in controlling DA release.

Finally, the cell body regions of DA neurons (VTA and substantia nigra *pars compacta*) show TSPO increases in the absence of amyloid plaques, thus confirming the inflammation observed in the midbrain of a mouse Alzheimer’s disease model.^4^ At the major DA neuron projection sites, we note an accumulation of amyloid deposits in a non-homogeneous manner. Indeed, while the accumbens is reached in its two sub-regions (core and shell), the caudate/putamen only accumulates plaques in the ventro-lateral sub-region. This predisposition of the accumbens vs the caudate/putamen for the Aβ deposits has also been described in humans.^10^^,^^80^ The increased sensitivity of the accumbens suggests that DA functions linked to the ventral striatum are preferentially affected as, for example, a dysfunction of reward circuits, an impairment of motivation or even the presence of anhedonia. Interestingly, all these dysfunctions have been observed in patients.^1^ The VTA-accumbens was also preferentially involved than substantia nigra-caudate/putamen axis in a mouse model of Alzheimer’s disease.^4^ In fact, in Tg2576 mice, selective apoptosis of neurons expressing the limiting DA synthesis TH enzyme within VTA has been reported.^4^ Thus, and in contrast to Parkinson's disease, which mainly affects the nigrostriatal pathway, our data in association with those in the literature assume an effect mainly on the mesolimbic pathway. The TgF344-AD rat therefore seems to represent a good model for studying the appearance of neurochemical alterations related to Alzheimer’s pathology within the DA system. Thus, our data suggest that the identification of pre-symptomatic functional alterations linked to the activity of D_2_R could perhaps represent a clinical diagnostic marker, and future studies in patients are necessary to confirm these observations.

As a whole, our observations demonstrate that the dopaminergic and the serotoninergic systems are functionally affected from the age of 6 months in TgF344-AD rats. A similar observation was reported concerning the activity of the norepinephrine system of the locus coeruleus.^23^ It is thus possible that alterations in functional interaction between monoamine systems can conduct to behavioural symptoms in Alzheimer’s disease as all of them take part in the regulation of cognitive processes.

## Conclusions

All of our approaches combining *in vivo* D_2/3_R availability estimation, *in vivo* translational DA release measures, behavioural tests of reactivity to stimulation of D_2_R and 5HT_2A_R, post-mortem density measurements agree to conclude that DA dysfunction and alteration in 5HT_2A_R−DA/D_2_R connectivity exist from the asymptomatic stages of Alzheimer-like pathology in the TgF344-AD rat. These functional alterations predispose not only the reduction of 5HT_2A_R density, but also the accumulation of TSPO-inflammation and amyloid plaques in DA system areas. These early events could turn out to be important since they also appear before alterations in spatial working memory, learning deficits and neurofibrillary tangles.^22^ Thus, the pre-symptomatic dysfunction in 5HT_2A_R−DA/D_2_R connectivity and D_2_R hyper-sensitivity in TgF344-AD rat make this Alzheimer’s disease model a choice for preclinical studies whose aim is to improve the early diagnosis of the pathology, but also to test the effectiveness of early therapy before the onset of irreversible disorders. Moreover, as it is now accepted that the presence of Aβ deposits cannot alone represent a diagnosis of Alzheimer’s disease as well as the presence of an upregulation of TSPO, our data support the idea that it would be important to test in MCI patients if impaired 5HT_2A_R−DA connectivity is also present and could therefore represent an earlier diagnostic tool for Alzheimer’s disease.

## Supplementary material

Supplementary material is available at *Brain Communications* online.

## Abbreviations

Acc =: accumbens core
Acs =: accumbens shell
AC =: auditory cortex
AD =: Alzheimer’s disease
CPU =: caudate/putamen
Cin =: cingulate cortex
DA =: dopamine
D_2_R =: dopamine D2 receptor
D_2/3_R =: dopamine D2−D3 receptor
dHipp =: dorsal hippocampus
DLS =: dorsolateral striatum
DMS =: dorsomedial striatum
EnC =: enthorinal cortex
MC =: motor cortex
5HT =: serotonin
5HT_2A_R =: serotonin 2A receptor
SNc =: substantia nigra pars compacta
SNr =: substantia nigra pars reticulata
SOM =: somatosensorial cortex
Sub =: subiculum
VC =: visual cortex
vHipp =: ventral hippocampus
VLS =: ventrolateral striatum
VTA =: ventral tegmental area
